# Anti‐inflammatory control of human skin keratinocytes by targeting nuclear transport checkpoint

**DOI:** 10.1002/ski2.356

**Published:** 2024-03-03

**Authors:** Yan Liu, Huan Qiao, Jozef Zienkiewicz, Jacek Hawiger

**Affiliations:** ^1^ Department of Medicine Division of Allergy Pulmonary and Critical Care Medicine Vanderbilt University School of Medicine Nashville Tennessee USA; ^2^ Department of Veterans Affairs Tennessee Valley Health Care System Nashville Tennessee USA; ^3^ Department of Molecular Physiology and Biophysics Vanderbilt University School of Medicine Nashville Tennessee USA

## Abstract

**Background:**

In the two common inflammatory skin diseases, Atopic Dermatitis (AD) and Psoriasis (Ps), keratinocytes (KCs) respond to immune insults through activation of proinflammatory transcription factors (TFs) and their translocation to the cell’s nucleus. Therein, the TFs induce expression of genes encoding mediators of skin inflammation. The Nuclear Transport Checkpoint Inhibitors (NTCIs) were developed to regulate nuclear translocation of activated TFs, the essential step of inflammatory response. This new class of cell‐penetrating peptide therapeutics controls inflammation caused by allergic, autoimmune, metabolic, and microbial insults. In preclinical model of AD, the treatment with NTCI, cSN50.1 peptide, suppressed the expression of Thymic Stromal Lymphopoietin (*TSLP*), the key gene in the development of allergic inflammation, among the 15 genes silenced by the NTCI. Here, we report the mechanism of anti‐inflammatory action of NTCI in human skin‐derived KCs.

**Objectives:**

We aimed to determine whether the NTCI treatment can protect human KCs from harmful inflammatory insults.

**Methods:**

Human primary KCs were pretreated with NTCI and challenged with the mix of cytokines Tumour Necrosis Factor alpha (TNF‐α) and Interleukin (IL)‐17A, or with Phorbol 12‐Myristate 13‐Acetate (PMA), and analysed for nuclear content of TFs and the expression of genes encoding mediators of inflammation.

**Results:**

The nuclear import of TFs, Nuclear Factor ĸB (NF‐ĸB) and Signal Transduction and Activator of Transcription 3 (STAT3), was inhibited in cells treated with NTCI. The expression of *TSLP,* along with genes encoding the core mediators of inflammation (*TNF*, *IL1B*, and *IL6*) was suppressed by NTCI. Noteworthy, NTCI silenced genes encoding Granulocyte‐Macrophage Colony‐Stimulating Factor (*CSF2*), and chemokine IL‐8 (*CXCL8*), responsible for skin infiltration by the eosinophils and other myelomonocytic cells.

**Conclusion:**

The control of inflammatory response in human KCs by NTCI is attributed to the inhibition of nuclear import of proinflammatory TFs. The protection of human KCs by NTCI, adds new perspectives to the completed Phase two clinical trial of the NTCI (AMTX‐100 CF) for AD (NCT04313400).

## Graphical abstract

Here we report previously unknown mechanism of the response of human primary, skin‐derived, keratinocytes (KCs) to proinflammatory insults. We found that the Nuclear Transport Checkpoint Inhibitor (NTCI), our Investigational New Drug, arrested the nuclear import of STAT3, the transcription factor involved in allergic inflammation, and the NF‐kappa B, prototypical proinflammatory Stress‐Responsive Transcription Factor, in human KCs. Among the genes and cognate proteins suppressed by the NTCI, was the major driver of allergic reactions, Thymic Stromal Lymphopoietin (TSLP), that promotes Th2 response. Moreover, other core proinflammatory mediators, TNF alpha, IL1 beta, IL6, IL8 and CSF2, were also suppressed in human KCs, thereby blocking the pathogenesis of AD and other dermatoses.

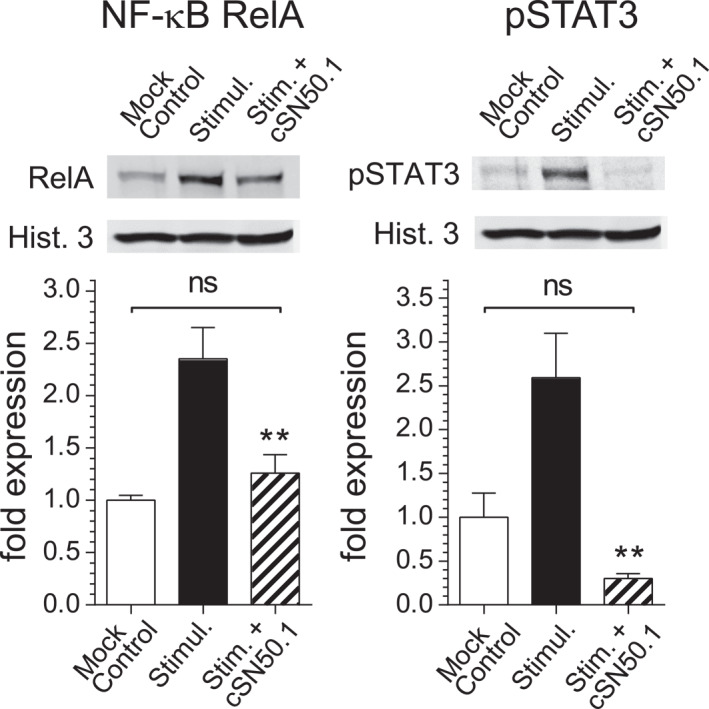

**What is already known about this topic?**

- We previously showed in preclinical model of Atopic Dermatitis (AD), aka Eczema, that a novel anti‐inflammatory and anti‐metabolic cell‐penetrating peptide, termed Nuclear Transport Checkpoint Inhibitor (NTCI), suppressed the expression of Thymic Stromal Lymphopoietin (TSLP), the key gene in keratinocytes (KCs) responsible for the development of allergic inflammation. Moreover, NTCI silenced the set of 15 genes encoding inflammatory mediators in the murine skin. However, it is unknown how the NTCI controls human KCs. As they guard the skin’s integrity by sensing the noxious inflammatory insults and producing multiple mediators of skin inflammation underlying AD, the mechanism of action of the NTCI in human KCs is unknown.

**What does this study add?**

- Here we analysed in human KCs the hitherto unknown mechanism of the nuclear translocation of the two proinflammatory transcription factors. We found that a new investigational drug, NTCI, arrested the nuclear import of STAT3, the transcription factor involved in allergic inflammation, as well as the NF‐κB, prototypical proinflammatory Stress‐Responsive Transcription Factor. Consequently, the NTCI silenced several proinflammatory genes, including TSLP, the major driver of the TH2 response, and the core mediators of skin inflammation. Thereby, the NTCI suppressed the production of their cognate proteins, TSLP, TNFα, IL1β, IL6, IL8 and CSF2, and blocked the key steps in the pathogenesis of Atopic Dermatitis.

## 1 INTRODUCTION

Keratinocytes (KCs) are strategically positioned in the outermost layer of the skin, the epidermis. They sense and respond to a wide range of biological, chemical and physical factors.^1^, ^2^ These insults evoke inflammatory response through the activation of the proinflammatory transcription factors (TFs). Their signalling to the cell’s nucleus is mediated by the nuclear transport adaptor proteins, importins (karyopherins) alpha and beta. In the nucleus, the translocated TFs activate the genes encoding the mediators of inflammation.^3^

Depending on the nature of an insult, the skin response leads to allergic, autoimmune, metabolic, microbial, or physical inflammation underlying a wide range of dermatologic diseases.^3^ The most common among them is Atopic Dermatitis (AD), aka eczema, globally afflicting an estimated 10%–20% of children and 5% of adults in all racial and ethnic groups.^4^, ^5^ AD, caused by allergic insults, is manifested by an intense itch, recurrent eczematous lesions, and a fluctuating course.^6^ In comparison, Psoriasis (Ps) is less common chronic, inflammatory skin disease of undetermined cause, displaying red, scaly plaques on the elbows, knees, scalp, and lower back, with possible involvement of any skin surface.^7^ Psoriasis is a largely T lymphocyte‐mediated disease in which activation of innate immune cells and pathogenic T cells leads to skin inflammation and hyperproliferation of KCs and their dysfunctional differentiation.^8^

We recently reported a new topical treatment of experimental AD by a Nuclear Transport Checkpoint Inhibitor (NTCI).^9^ The NTCI is the cell‐penetrating peptide, which simultaneously targets two nuclear transport shuttles, cytoplasmic adaptor proteins termed importin α5 and importin β1.^3^, ^10^, ^11^ These two cytoplasmic proteins are required for the nuclear translocation of an entire set of proinflammatory Stress‐Responsive Transcription Factors (SRTFs), for example, Nuclear Factor kappa B (NF‐ĸB) and Nuclear Factor of Activated T cells (NFATs), and Metabolic Transcription Factors (MTFs), for example, Sterol Regulatory Element‐Binding Proteins (SREBPs) 1 and 2. By preventing their nuclear transport, the NTCI disables the inflammatory regulome thereby silencing at least 54 genes encoding the mediators of inflammation and apoptosis in allergic, autoimmune, constitutive, metabolic, microbial, and physical inflammatory responses.^3^, ^12^ Strikingly, in experimental sepsis, the NTCI controlled 3735 sepsis‐induced genes in the lungs and 1951 sepsis‐induced genes in the kidneys.^13^ The remaining nuclear transport adaptor proteins, importins alpha (α1, α3, α4, α6, and α7), which are not targeted by the NTCIs, can still ferry other nuclear proteins, including different TFs and histones, during the NTCI‐imposed selective nuclear blockade.

In allergic inflammation, epidermal KCs fire up the *TSLP* gene that encodes the Thymic Stromal Lymphopoietin (TSLP) protein. The TSLP evokes the Th2 response underlying the mechanism of AD^14^, ^15^ and is also responsible for inducing itch, the pathognomonic sign of AD.^16^ Knocking out the gene encoding TSLP prevented the development of AD.^17^ Therefore, we aimed to establish in the cultured human KCs: (i) whether suppression of *TSLP* gene by the NTCI depends on the inhibition of the nuclear translocation of the two key TFs in allergic inflammation, NF‐ĸB RelA and STAT3, and (ii) whether other genes that encode the mediators of the acute phase protein response, microvascular leak, redness, and infiltration by inflammatory cells, are also silenced in human KCs by NTCI. These genes are involved in AD and other human inflammatory skin diseases.^9^

Henceforth, we analysed nuclear signalling by TFs at the subcellular level in primary human KCs derived from adult skin to determine whether their broad inflammatory response^2^ is mediated by the two acute phase response TFs, NF‐κB Rel A and STAT3.^18^, ^19^ We reasoned that the NTCI, known to arrest the nuclear translocation of TFs responding to inflammatory stress in immune and non‐immune cells,^3^ should be similarly effective in cultured human KCs.

## 2 MATERIALS AND METHODS

### 2.1 The synthesis and purification of the cell‐penetrating Nuclear Transport Checkpoint Inhibitor, cSN50.1 peptide

Cell‐penetrating NTCI peptide, cSN50.1 (AAVALLPAVLLALLAPCVQRKRQKLMPC, 2986 Da) was synthesised as described elsewhere.^20^ Briefly, the peptide chain was assembled through Solid Phase Peptide Synthesis (SPPS) according to standard Fmoc chemistry protocols using an automated peptide synthesiser FOCUS XC (AAPPTec, Louisville, KY, USA). Crude peptides were removed from the resin with a TFA cleavage cocktail and purified by dialysis against double‐distilled water in 1 KDa membrane (Spectra/Por 7; Spectrum Laboratories, Rancho Dominguez, CA). The purity and structure of the final products were verified respectively by an analytical C18 reversed phase high‐performance liquid chromatography (RP HPLC; Beckman Coulter GOLD System, Brea, CA, USA) and MALDI mass spectroscopy (Voyager Elite; PerSeptive Biosystems, Framingham, MA, USA).

### 2.2 Cell culture and treatment

Human Primary Epidermal Keratinocytes (ATCC; Manassas, VA, USA) were cultured according to the supplier’s recommendations in 10 cm dishes until 80 % confluent. Cells were stimulated with the mixture of 10 ng/mL TNF‐α together with 20 μg/ml IL‐17A, and or with 50 nM Phorbol 12‐Myristate 13‐Acetate (PMA) (all from Millipore‐Sigma, Burlington, MA, USA) and incubated at 37 °C in 5 % CO_2_. Thirty minutes before the challenge, cells were pretreated with the NTCI (cSN50.1 peptide), 10 μM (for KCs stimulated with TNF‐α/IL‐17A) or 30 μM (for KCs stimulated with PMA). The samples of the cell‐free supernatant for the determination of the cytokines and chemokines were collected at 24‐ and 48‐h intervals after TNF‐α/IL‐17A, or at 6‐ and 24‐h intervals post PMA challenge.

### 2.3 The nuclear translocation of SRTFs in cultured human keratinocytes

The KCs were harvested 48 h after TNF‐α/IL‐17A stimulation, lysed with a hypotonic buffer^20^ containing 2 % NP‐40, protease and phosphatase inhibitors (Roche, Indianapolis, IN, USA), and washed 3 times to yield clean nuclei. Nuclear proteins were obtained by a high‐salt extraction (450 mM NaCl; 4 °C, 2000 rpm 30 min). The TNF‐α/IL‐17A‐stimulated KCs not treated with the NTCI and unstimulated cells (mock control) served as positive and negative controls, respectively. In all conditions, the cell viability was greater than 80 %.

The nuclear content of the 2 TFs, NF‐ĸB RelA and phosphorylated STAT3 (pSTAT3) was determined by quantitative immunoblotting using a rabbit monoclonal anti‐NF‐κB p65 (RelA) antibody and rabbit polyclonal anti‐pSTAT3. The rabbit monoclonal anti–Histone 3 (all from Cell Signalling Technology, Danvers, MA, USA) was used to measure Histone 3 as a nuclear loading control for normalisation. Li‐Cor IRDye fluorescently labelled secondary antibodies were used for detection of immunoreactive bands. Immunoblots were analysed on a Li‐Cor Biosciences Odyssey Infrared Imaging System and bands were quantified by densitometry analysis using LI‐COR Image Studio 3.1 (Lincoln, NE, USA). Each cell‐based experiment was performed in duplicate and repeated at least twice to assure experimental significance and reproducibility.

### 2.4 Gene expression assay by real‐time quantitative reverse transcription PCR

Cultured human primary KCs were disrupted in the lysis buffer on ice 48 h post TNFα/IL‐17A challenge. Total RNA was isolated using NucleoSpin RNA Plus kit (Macherey‐Nagel, Düren, Germany) according to the manufacturer’s instructions. RNA concentration and purity were determined using a NanoDrop One spectrophotometer (Thermo Fisher Scientific, Waltham, MA, USA). One μg of the obtained RNA was reverse transcribed using an iScript cDNA synthesis kit (Bio‐Rad, Hercules, CA, USA). A real‐time quantitative reverse transcription polymerase chain reaction (qRT PCR) was carried out in a 96‐well plate on a QuantStudio 3 instrument using the Taqman Fast Advanced Master Mix and FAM‐labelled probes of analysed genes (all from Thermo Fisher Scientific, Waltham, MA, USA) according to the manufacturer’s protocol. The raw Ct values were converted into relative expression levels using Livak’s methods (2^−∆∆Ct^) with either αTubulin gene as a reference, and the Mock Control group as a calibrator (control). Converted Ct values were used for statistical analysis. The qRT PCR measurement was performed twice in duplicate to ensure reproducibility and statistical significance.

### 2.5 Cytokine/chemokine proteins assay

Cytokines and chemokines produced by the stimulated human KCs were measured in a medium collected from the cultured cells at indicated timepoints. Determination of the TSLP protein level was performed by enzyme‐linked immunosorbent assay (ELISA) (Invitrogen, Carlsbad, CA, USA). The levels of IL‐6, TNF‐α, GM‐CSF (CSF2) and chemokine IL‐8 (CXCL8) proteins were determined by the cytometric bead array (CBA) assay (BD Biosciences, Franklin Lakes, NJ, USA) in the Vanderbilt University Medical Centre Flow Cytometry Shared Resource. The cytokine/chemokine assay was performed twice in duplicate to ensure reproducibility statistical significance.

### 2.6 Statistical analysis

The normal distribution of data sets was verified using a normal probability plot (q‐q) and a Kolmogorov‐Smirnov Normality Test. A statistical analysis was performed using tools built‐in Prism 6 software (GraphPad, La Jolla, CA, USA). Immunoblots of SRTFs (NF‐κB RelA and pSTAT3) in nuclear extracts from cultured primary human KCs, and gene expression profile in human KCs were analysed by ordinary one‐way ANOVA with an uncorrected Fisher’s LSD test for a multiple comparison. The levels of proteins representing cytokines TSLP, IL‐6, TNF‐α, GM‐CSF, and chemokine IL‐8 in the medium from cultured human KCs were evaluated by the repeated measure two‐way ANOVA (analysis of variance) using an uncorrected Fisher’s LSD test for multiple comparison. The data is presented as a mean ± S.E.M. (standard error of the mean). *p* values of <0.05 were considered significant.

## 3 RESULTS

### 3.1 Cultured human KCs respond to proinflammatory cues by dispatching the SRTFs, NF‐ĸB RelA and STAT3, to the nucleus

The human KCs respond to the proinflammatory insults through the activation of signalling pathways that translocate SRTFs to the cell’s nucleus. Therein, these proinflammatory SRTFs activate the promoters and enhancers of the genes encoding the mediators of inflammation.^3^

**FIGURE 1:**
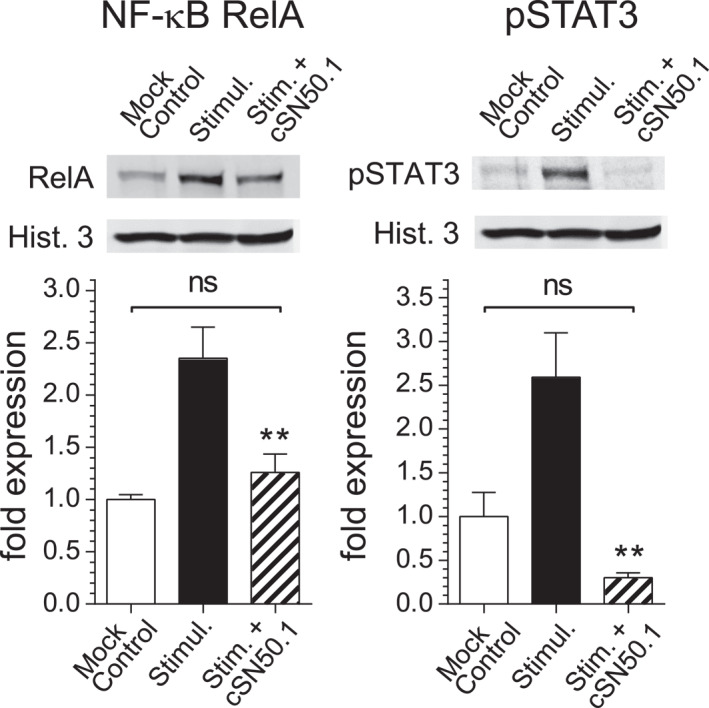
NTCI treatment of the human primary KCs inhibits proinflammatory signalling to the nucleus through reducing the nuclear translocation of NF‐κB RelA and STAT3. The human primary KCs were pretreated with 10 μM NTCI (cSN50.1 peptide) 30 min before stimulation with a mix of two cytokines: TNF‐α (10 ng/ml) and IL‐17A (20 μg/ml)^2^ and incubated overnight. The nuclear content of the two SRTFs, NF‐κB RelA and pSTAT3 (phosphorylated STAT3), was determined by a quantitative immunoblotting analysis using the LI‐COR Odyssey Infrared Imaging System. The data is presented as a mean + S.E.M. (*n* = 4). The statistical significance of a difference between the nuclear content of two SRTFs in the TNF‐α/IL‐17A‐stimulated cells (Stimulated) and stimulated cells treated with the NTCI, cSN50.1 peptide (Stim. + cSN50.1) was determined by an ordinary one‐way ANOVA with uncorrected Fisher’s LSD test for multiple comparison, **p < 0.005 (the unedited full‐length immunoblots are presented in the Supplementary Figure S1). KCs, keratinocytes; NF‐ĸB, Nuclear Factor kappa B; NTCI, Nuclear Transport Checkpoint Inhibitor; SRTFs, Stress‐Responsive Transcription Factors; STAT3, Signal Transduction and Activator of Transcription 3; TNF‐α, Tumor Necrosis Factor alpha.

Hence, we analysed the nuclear signalling at the subcellular level in the primary human KCs derived from adult skin to determine whether the two acute phase response TFs, NF‐κB RelA and STAT3,^18^, ^19^ can be controlled by the NTCI. Indeed, the NTCI reduced the nuclear translocation of both NF‐ĸB and STAT3 in response to proinflammatory agonists, as compared to the unstimulated cells (Figure 1).

We showed that the NTCI controlled proinflammatory nuclear signalling mediated by NF‐κB RelA and pSTAT3, among other SRTFs activated by the cytokine mix of TNF‐α/IL‐17A in cultured human KCs.

### 3.2 The NTCI suppressed expression of genes encoding the key inflammatory mediators in human KCs

**FIGURE 2:**
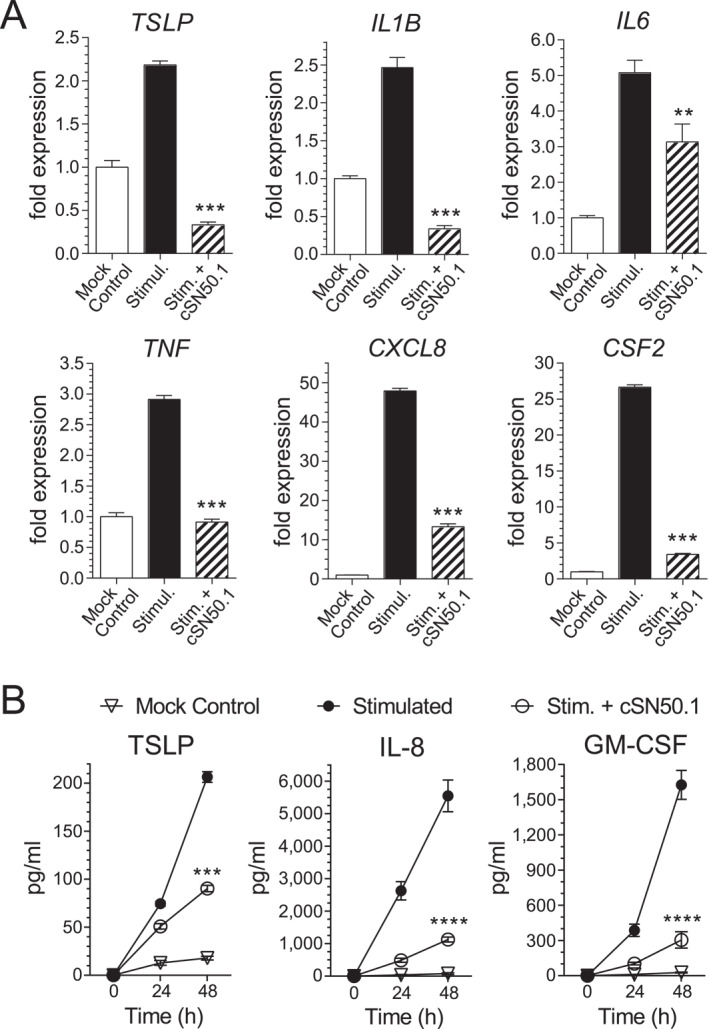
NTCI treatment inhibits expression of TSLP and other proinflammatory genes encoding proteins that mediate skin inflammation in cultured human KCs. (a) NTCI suppresses genes encoding the inflammatory mediators in cultured human primary KCs stimulated with TNF‐α/IL‐17A. The gene expression was determined using real‐time quantitative reverse transcription PCR (qRT PCR). The relative expression levels were established with Livak’s methods (2^−∆∆Ct^) with αTubulin gene as the reference and a Mock Control group as the calibrator. The data is presented as a mean + S.E.M. (*n* = 4). The statistical significance of the difference between the transcript levels in the cells Stimulated untreated and cells Stimulated treated with the NTCI, cSN50.1 peptide was determined using an ordinary one‐way ANOVA with an uncorrected Fisher’s LSD test for a multiple comparison, **p < 0.005, ***p < 0.0005. (b) NTCI suppresses production of cognate proteins, TSLP, GM‐CSF (CSF2) and IL‐8 (CXCL8) in cultured human primary KCs stimulated with TNF‐α/IL‐17A. Supernatant samples were collected from the medium of cultured KCs at the indicated timepoints after TNF‐α/IL‐17A stimulation. The proteins concentration was determined in the culture medium using ELISA (TSLP) and CBA (GM‐CSF and IL‐8) assays. The data is presented as mean ± S.E.M. (*n* = 4). The statistical significance of the difference between the cytokine/chemokine levels in the media from stimulated cells and stimulated cells treated with the NTCI, cSN50.1 peptide, was determined by a repeated measure two‐way ANOVA using an uncorrected Fisher’s LSD test for multiple comparison, ***p < 0.0005, ****p < 0.0001. CSF2, Granulocyte‐Macrophage Colony‐Stimulating Factor; CXCL8, chemokine IL‐8; KCs, keratinocytes; NTCI, Nuclear Transport Checkpoint Inhibitor; TNF‐α, Tumor Necrosis Factor alpha; TSLP, Thymic Stromal Lymphopoietin.

The inhibition of nuclear translocation of the two SRTFs, NF‐κB RelA and pSTAT3, in human KCs (see Figure 1) underlied the silencing of the key genes mediating AD and other inflammatory skin diseases. Of special importance is the suppression of the gene encoding TSLP (Figure 2a). This cytokine is essential for the development of AD by eliciting the Th2 response.^14^, ^15^ The *TSLP* gene knock out prevented calcipotriol‐induced AD.^17^ Generally, TSLP mediates type 2 immunity at barrier surfaces and has been linked to the widespread allergic and inflammatory diseases of the skin (eczema), airways (asthma), and gut (eosinophilic oesophagitis).^21^ The expression of TSLP‐encoding gene in allergic diseases is regulated by the NF‐ĸB and STAT3.^18^, ^19^

Arresting the nuclear translocation of NF‐κB RelA and STAT3, among other SRTFs and MTFs^3^ in human KCs by the NTCI (see above Figure 1), prevented activation of a spectrum of other inflammatory genes encoding crucial mediators of AD. Besides TSLP, they included the genes encoding three acute phase inflammatory mediators, *IL1B*, *IL6*, and *TNF* (see Figure 2a). We also found that NTCI suppressed the genes encoding GM‐CSF (*CSF2*) and IL‐8 (*CXCL8*), which are known contributors to the mobilisation and trafficking of eosinophils, monocytes/macrophages, and other myelomonocytic cells in dermal infiltrate.^22^, ^23^

As expected, the NTCI comparably suppressed the protein levels of TSLP, GM‐CSF, and IL‐8 in the human KCs, as measured in the culture medium (Figure 2b). Thus, the decreased protein levels of GM‐CSF and IL‐8 were consistent with their transcripts reduced by the NTCI (Figure 2) reflecting their cognate gene suppression in the human KCs. Together, the concordance of the genomic analysis with the proteomic analysis of selected mediators of inflammation in the culture medium reaffirmed the inhibitory action of the NTCI.

### 3.3 The NTCI protects human KCs challenged with Phorbol 12‐Myristate 13‐Acetate (PMA), another inducer of the genes encoding key inflammatory mediators

**FIGURE 3:**
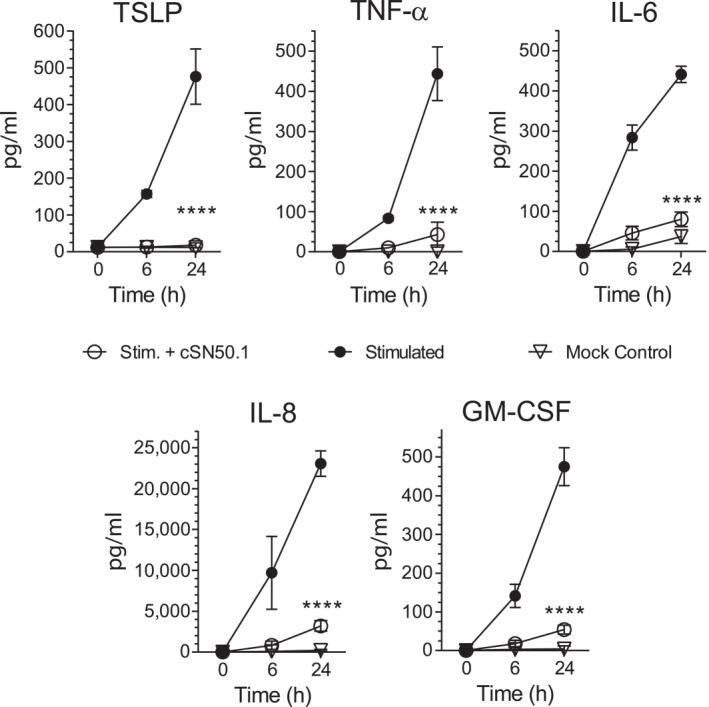
NTCI treatment suppresses time‐dependent production of cytokines and chemokines in cultured human KCs stimulated with PMA. The human primary KCs derived from adult skin were pretreated with NTCI (cSN50.1 peptide) 30 min before stimulation with PMA. Supernatant samples were collected from the medium of cultured KCs at the indicated timepoints after PMA challenge. The proteins concentration in the culture medium were determined using ELISA (TSLP) and CBA (TNF‐α IL‐6, GM‐CSF, and IL‐8) assays. The data is presented as mean ± S.E.M. (*n* = 4). The statistical significance of the difference between the cytokine/chemokine levels in the media from stimulated cells and stimulated cells treated with the NTCI, cSN50.1 peptide, was determined by a repeated measure two‐way ANOVA using an uncorrected Fisher’s LSD test for multiple comparison, ****p < 0.0001. CBA, cytometric bead array; ELISA, enzyme‐linked immunosorbent assay; KCs, keratinocytes; NTCI, Nuclear Transport Checkpoint Inhibitor; PMA, Phorbol 12‐Myristate 13‐Acetate; TNF‐α, Tumor Necrosis Factor alpha; TSLP, Thymic Stromal Lymphopoietin.

We also tested the action of the NTCI on human KCs’ response to another inducer of skin inflammation, Phorbol 12‐Myristate 13‐Acetate (PMA). PMA causes chronic inflammatory process mediated by Kit, a receptor tyrosine kinase, required for the mast cell accumulation in the skin.^24^ As anticipated, the PMA evoked the time‐dependent production of inflammatory mediators, TSLP, IL‐8, GM‐CSF, TNF‐α, and IL‐6, in the human KCs. These inflammatory proteins were also suppressed by the NTCI as compared to the control, untreated human KCs (Figure 3).

Altogether, we detected an increased nuclear translocation of both TFs, NF‐κB RelA and STAT3, in response to stimulation by the inflammatory cytokine duo of TNF‐α and IL‐17A or by PMA. The cell‐penetrating NTCI, cSN50.1 peptide, significantly reduced the nuclear translocation of NF‐κB RelA and STAT3 in human KCs (Figure 1). Subsequently, we found that the NTCI suppresses the production of TSLP in human KCs. Therein, the NTCI also controls the expression of five other genes encoding the mediators of skin inflammation, including cytokines IL1β, IL6, *TNF*, chemokine *CXCL8* (aka IL8), and Colony Stimulating Factor 2 (*CSF2* aka Granulocyte Monocyte CSF). Thus, the NTCI, a novel broad‐spectrum anti‐inflammatory agent protects human KCs at the nuclear transport level potentially averting a range of human inflammatory skin diseases. The translational value of NTCI is being evaluated in an completed Phase two Clinical Trial for mild to moderate AD (NCT04313400).

## 4 DISCUSSION

Recently, we reported the NTCI, cell‐penetrating cSN50.1 peptide, as a novel topical agent for the treatment of AD, a recurrent inflammatory skin disease afflicting a sizeable population of children and adults worldwide.^9^ We unravelled the genomic mechanism of experimental AD, which was induced by a repeated challenge with MC903 (calcipotriol) for 23 days. This vitamin D_3_ analog was found to cause AD during its clinical trial in patients with psoriasis.^25^ Importantly, mice deficient in the gene encoding TSLP were resistant to the induction of AD by MC903.^17^ The key role of TSLP in AD as a driver of Th2‐mediated allergic inflammation has been previously elucidated.^14^, ^15^

Here, we provided a new line of evidence for human KCs’ activation, their transcriptional mechanism, and its control by the NTCI through the inhibition of the nuclear translocation of the two acute phase response TFs, NF‐κB Rel A and STAT3. These 2 TFs are linked to the Endoplasmic Reticulum (ER) stress.^18^, ^19^ The inhibition of these two key TFs in human KCs correlated with the significant silencing of at least six genes encoding TSLP and other major mediators of skin inflammation.

The striking suppression of *TSLP* gene in human KCs (see Figure 2), linked to the inhibition of the nuclear translocation of NF‐κB RelA and STAT3 (see Figure 1), suggests the itch‐reducing potential of the NTCI. Both, importin α5 (directly targeted by the NTCI), and STAT3 (unable to bind to its nuclear shuttle in the presence of the NTCI), are linked to the injury of peripheral nerves.^26^ This pathogenetic association may also contribute to the itch‐reducing action of the NTCI.

The NTCI also suppressed the genes encoding three acute phase inflammatory mediators, IL1β, IL6, and *TNF* (Figure 2a). It is noteworthy that all of them belonged to the set of 15 genes silenced by the NTCI in the murine skin studied in the preclinical model of AD.^9^ In the human KCs, we also found that the NTCI suppressed the genes encoding GM‐CSF (*CSF2*) and IL‐8 (*CXCL8*), which mediate skin infiltration by eosinophils, monocytes/macrophages, and other myelomonocytic cells, in addition to the previously observed suppression of the CD4+ T cells in the skin infiltrate.^9^ Proteomic analysis of human KC’s culture medium was concordant with the gene expression induced in human KCs by TNF‐α/IL‐17A.

Cumulatively, the hitherto unreported suppression of the *TSLP* gene in human KCs by the NTCI offers a new targeting angle to the control of AD and other human skin diseases. They include the Netherton Syndrome due to overexpression of *TSLP* ^27^ and an autoinflammatory disorder of the skin and bones caused by the constitutive overproduction of IL‐1β.^28^ In addition, the three other genes expressed in activated human keratinocytes, IL1β, IL6, and *TNF* (Figure 2) evoke the “acute phase protein response” associated with the ER stress.^3^, ^18^, ^29^ The suppression of these three mediators by the NTCI would counteract both the localised and potentially systemic inflammatory response in AD and other skin diseases.

**FIGURE 4:**
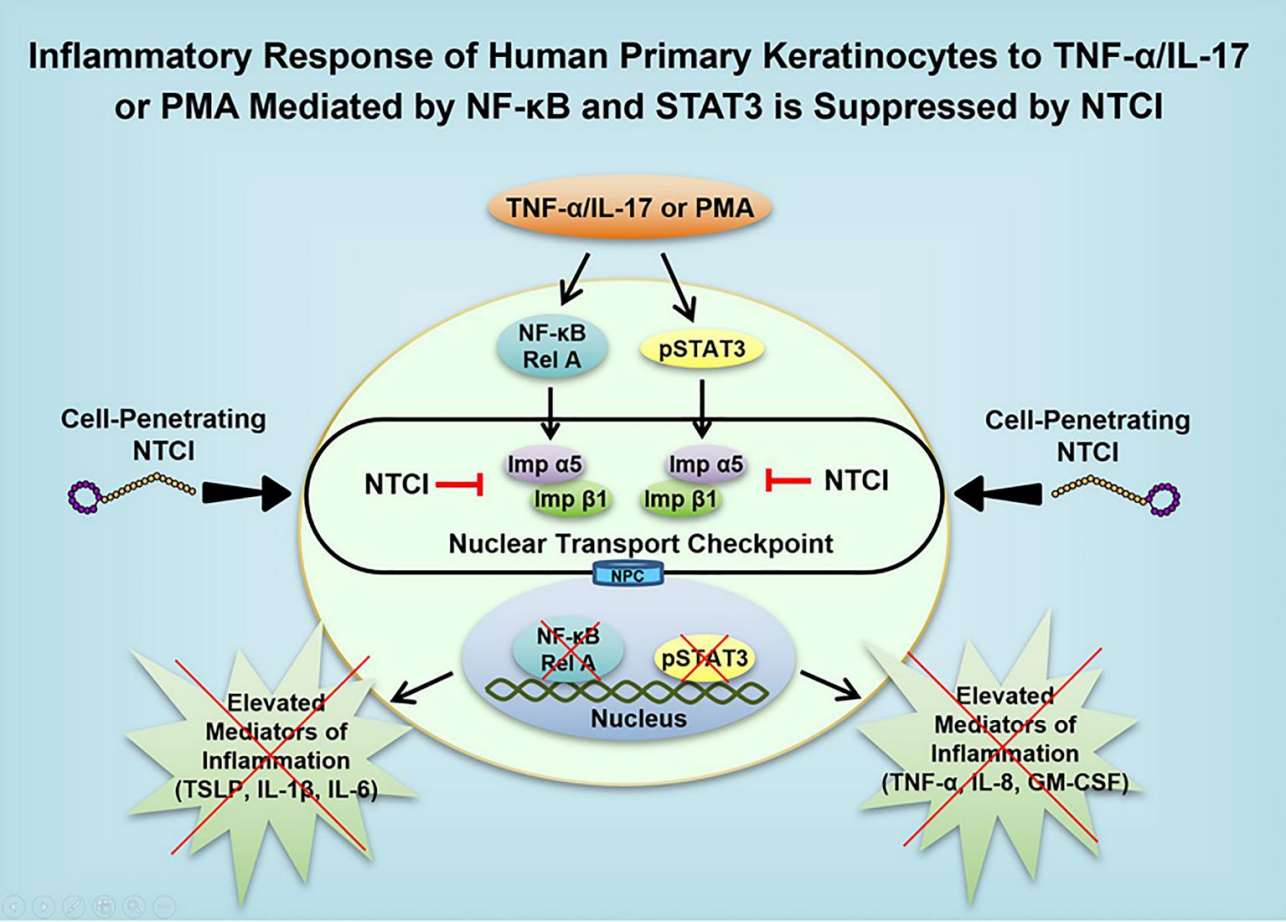
The mechanism of the NTCI cytoprotective action in inflamed human primary KCs. A mix of cytokines TNF‐α and IL‐17A or skin irritant, PMA, activate the proinflammatory signalling pathways. These pathways are transduced by two SRTFs, NF‐κB RelA and pSTAT3, known to activate proinflammatory and proapoptotic genes. The nuclear translocation of NF‐κB RelA and pSTAT3 is mediated by a nuclear import adaptor complex, Imp α5/Imp β1. The NTCI (cell‐penetrating cSN50.1 peptide) controls this checkpoint. Consequently, selective signalling to the nucleus by SRTFs is stopped and the activation of genes encoding the mediators of inflammation and apoptosis is suppressed [see Suppl. Figure S1 in reference^9^] for more details of this and other signalling pathways as well as the points of action of other anti‐inflammatory therapeutics. KCs, keratinocytes; NF‐ĸB, Nuclear Factor kappa B; NPC, Nuclear Pore Complex; NTCI, Nuclear Transport Checkpoint Inhibitor; PMA, Phorbol 12‐Myristate 13‐Acetate; pSTAT3, phosphorylated STAT3; SRTFs, Stress‐Responsive Transcription Factors; SRTFs, Stress‐Responsive Transcription Factors; TNF‐α, Tumor Necrosis Factor alpha.

The discovery of the role of nuclear transport pathway in the transcriptional control of the key genes in human KCs is of broader significance. Our results explain why the expression of at least 33 inflammatory mediators in the human KCs^2^ poses a major challenge to the currently used methods of treatment that target only one or two protein mediators of AD. Since AD is chiefly mediated by the proinflammatory SRTFs, encompassing NF‐ĸB and pSTAT3, as depicted in Figure 4, as well as AP1 (cFos and cJun), STAT1, and NFATs,^3^ the NTCI controls all of them thereby offering a new and unique treatment strategy for AD and other inflammatory skin diseases.

The NTCI inhibits the nuclear translocation of only larger TFs (MW > 45 kDa), such as SRTFs and MTFs ferried by the Imp α5/Imp β1 complex (see supplemental Figure S1 in reference 9). Importantly, the smaller TFs (MW < 45 kDa), which are essential to the cell survival and maintenance, can freely translocate to the nucleus to contribute to the homeostasis and lifespan of cells in the presence of the NTCI.^11^, ^12^ The remaining nuclear transport adaptor proteins, other than Imp α5/Imp β1complex, namely, importins alpha (α1, α3, α4, α6, and α7), which are not targeted by the NTCIs, can still ferry other nuclear proteins, including different TFs and histones, during the NTCI‐imposed selective nuclear blockade. Hence, we found that the NTCI treatment did not alter the expression of the five housekeeping genes (*Gusb, Hprt1, Hsp90ab1, Gapdh,* and *Actb*).^30^

The NTCI reported in this study represents a new class of broad‐spectrum anti‐inflammatory agents for topical (localised) and, if needed, systemic therapy. Other broad‐spectrum anti‐inflammatory agents, the glucocorticoids, target the inflammatory regulome via the action of the cognate nuclear receptor, which functions as a transcription factor. Inadvertently, this mode of glucocorticoids’ action causes skin atrophy, osteoporosis, immunosuppression, and dysregulated metabolism (hyperglycemia, hyperlipidaemia).^3^ In striking contrast, the NTCI reduces blood glucose, cholesterol, and triglycerides by controlling the nuclear translocation of metabolic TFs, SREBP1and SREBP2, while increasing the innate immunity‐mediated clearance of bacteria through the inhibition of nuclear transport of SRTFs.^3^, ^31^ Thus, the simultaneous control of nuclear translocation of SRTFs and SREBPs potentially enables a single NTCI, that is, cSN50.1 peptide, to counteract AD associated with Metabolic Syndrome in paediatric and adult patients.^32^ In response to fatty acids, the CD36‐SREBP1 signalling activates TSLP in mice.^33^ We found that the NTCI inhibits nuclear translocation of SREBP1.^11^ Moreover, the paediatric patients suffer AD often complicated by skin infections.^34^, ^35^ They may additionally benefit from the NTCI that counteracted bacterial dissemination in sepsis^13^ and suppressed Staphylococcal Enterotoxin B‐induced inflammation in the lungs.^36^

While our study of cultured human skin‐derived primary KCs is limited to their monolayers, they form the complex multilayered barrier in the skin.^37^ Such a skin‐based structure was studied by us in the experimental model of AD induced by MC903 (calcipotriol) for 23 days.^9^ Therein, the NTCI treatment suppressed skin infiltration by eosinophils, macrophages, and T cells. Strikingly, the proliferation of Ki67‐positive cells in the basal zone of the epidermis was also attenuated by the NTCI. Some of these findings can be extended to the in vitro 3D models of human KCs in an environment similar to human skin, and to KCs obtained from patients with AD and Ps.

Taken together, the human KCs respond to noxious allergic, metabolic, and microbial proinflammatory stimuli in three steps: (1). Activation of SRTFs, that encompass NF‐ĸB and STAT3, among others; (2). Nuclear translocation of NF‐ĸB and STAT3 and other SRTFs by importin α5—importin β1 complex to activate inflammatory regulome in the cell’s nucleus (see Suppl. Figure S1 in Ref. 9) whereas SREBPs are translocated by importin β1 also targeted by the NTCI; and (3). Activation and expression of the multiple genes encoding inflammatory mediators, cytokines, chemokines, hematopoietic and vascular growth factors, intracellular signal transducers, cell‐adhesion molecules, and metabolic mediators.^3^

The NTCI protects primary human skin‐derived KCs from the noxious inducers of skin inflammation through the transcriptional inhibition of the key genes involved in AD and other inflammatory skin diseases. Hence, their silencing in human KCs paves the way for the effective new treatment of AD and potentially Ps, the two prominent inflammatory skin diseases in the world.^4^, ^5^ Likewise, other common and rare inflammatory skin diseases are potential candidates for treatment with the NTCI. They include: (i) injury of KCs by the UV and gamma radiation^1^ that activates TFs NF‐κB and AP1^38^; (ii) auto‐inflammatory skin diseases, such as the uncontrolled production of IL‐1β in the constitutive disorder of the skin and bones,^28^ (iii) an excessive expression of *TSLP* in the Netherton Syndrome,^27^ and (iv) early severe allergic disease resulting from the inborn errors of immunity due defective TFs.^39^

Cumulatively, our study of the cytoprotection of the human KCs by the selective nuclear blockade with the NTCI, adds a new and broad translational perspective to the completed Phase two clinical trial of the NTCI (AMTX‐100 CF) for mild to moderate AD (NCT04313400).

## CONFLICT OF INTEREST STATEMENT

YL, JZ, HQ, and JH are co‐inventors of patents assigned to Vanderbilt University and the US Department of Veterans Affairs. JH co‐founded Amytrx Therapeutics, Inc.

## AUTHOR CONTRIBUTIONS

**Yan Liu**: Data curation (equal); Investigation (equal); Methodology (equal); Validation (supporting); Writing—original draft (supporting). **Huan Qiao**: Data curation (equal); Investigation (equal); Methodology (supporting); Validation (supporting); Writing—original draft (supporting). **Jozef Zienkiewicz**: Conceptualisation (equal); Data curation (equal); Formal analysis (lead); Investigation (equal); Methodology (equal); Project administration (equal); Resources (equal); Software (lead); Supervision (equal); Validation (equal); Visualisation (lead); Writing—original draft (supporting); Writing—review & editing (equal). **Jacek Hawiger**: Conceptualisation (equal); Funding acquisition (lead); Methodology (equal); Project administration (equal); Resources (equal); Supervision (equal); Validation (equal); Writing—original draft (lead); Writing—review & editing (equal).

## ETHICS STATEMENT

Human Primary Epidermal KCs (ATCC; PCS‐200‐011) used in this study were purchased from American‐Type Culture Collection. Ethical approval and consent for the use of these cells are not required in accordance with local/national guidelines.

## Supporting information

Supplementary Material

## Data Availability

All data generated or analyzed during this study are included in this published article and its supplementary information files.
