# Supplementary material for: Anti‐inflammatory control of human skin keratinocytes by targeting nuclear transport checkpoint
Source: Skin Health Dis. 2024 Mar 3;4(3):e356. doi: 10.1002/ski2.356 (PMC11150741; doi:10.1002/ski2.356)
Supplement: Supplementary file 1 — Supplementary Material [file SKI2-4-e356-s001.docx]

**Supplementary Materials**

**Anti-inflammatory Control of Human Skin Keratinocytes by Targeting Nuclear Transport Checkpoint**

Yan Liu^1,2,#^, Huan Qiao^1,#^, Jozef Zienkiewicz^1,2,#^, and Jacek Hawiger^1,2,3,^*

^1^ Vanderbilt University School of Medicine, Department of Medicine, Division of Allergy, Pulmonary and Critical Care Medicine, Nashville, Tennessee, United States of America

^2^ Department of Veterans Affairs, Tennessee Valley Health Care System, Nashville, Tennessee, United States of America

^3^ Vanderbilt University School of Medicine, Department of Molecular Physiology and Biophysics, Nashville, Tennessee, United States of America

^#^ Y.L., H.Q., and J.Z. contributed equally to this study.

* To whom correspondence should be addressed:

Jacek Hawiger

Vanderbilt University Medical Center

21^st^ Avenue South, T-1218, MCN

Nashville, TN 37232, USA

phone: +1 (615) 828-8718

e-mail: jack.hawiger@vumc.org

**Suppl. Fig. S1. Unedited full-length immunoblots used for preparation of Figure 1A.**

Immunoblot analysis of NF-κB RelA and pSTAT3 in nuclear fractions of primary human KCs stimulated with TNF-α and IL-17A.


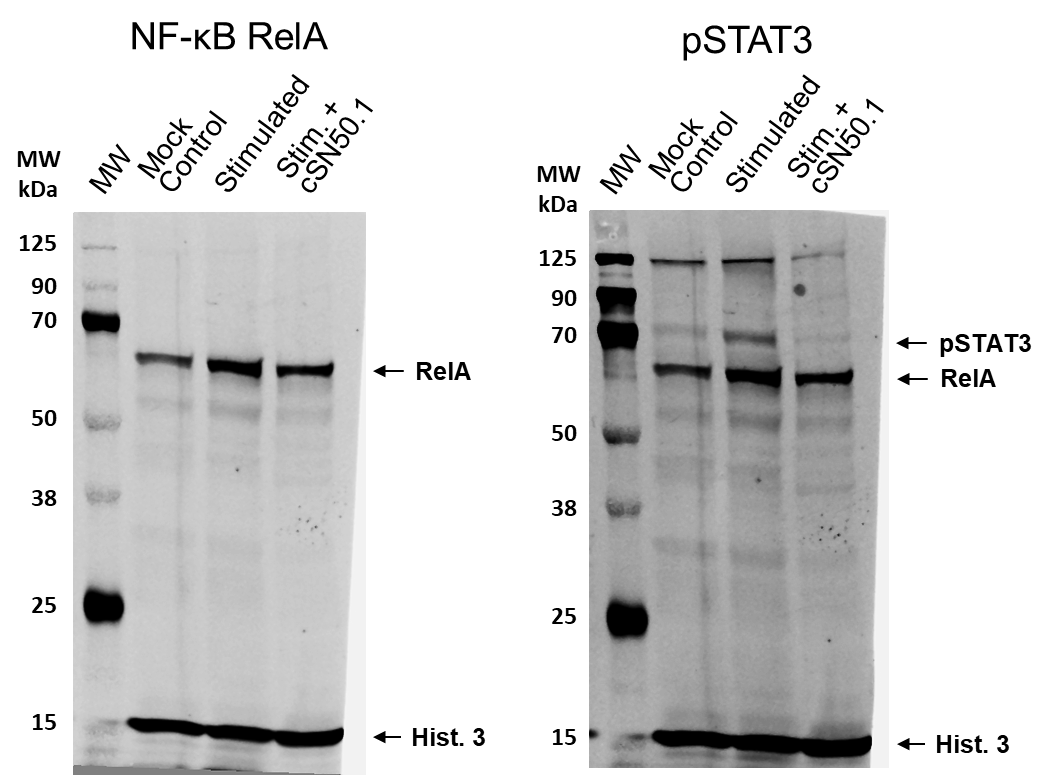


SDS PAGE electrophoreses were performed on 10% gels. Protein samples were transferred into cellulose membranes using Trans-Blot Turbo System (Bio-Rad, Hercules, CA, USA). Membranes were immunoblotted with rabbit polyclonal anti-Histone 3 primary antibody (and rabbit monoclonal anti-NF-κB p65 (RelA) antibody (both from Cell Signaling Technology, Danvers, MA, USA) following blotting with goat anti-rabbit IRDye 680RD (LI-COR, Lincoln, NE, USA) secondary antibodies for detection in 700 nm channel. After immunoblotting was completed, membranes were scanned with 700 nm channel on Odyssey CLx Infrared Imaging System (LI-COR, Lincoln, NE, USA). The membrane was then reblotted with rabbit polyclonal anti p-STAT3 (Cell Signaling Technology, Danvers, MA, USA) following blotting with goat anti-rabbit IRDye 680RD (LI-COR, Lincoln, NE, USA) secondary antibodies. The membrane was then rescanned using the Odyssey CLx Infrared Imaging System. Images were quantitatively analyzed using Image Studio 3.1 software (Li-COR, Lincoln, NE, USA).

**Suppl. Fig. S2. Viability of cultured Keratinocytes in experimental settings.**

**
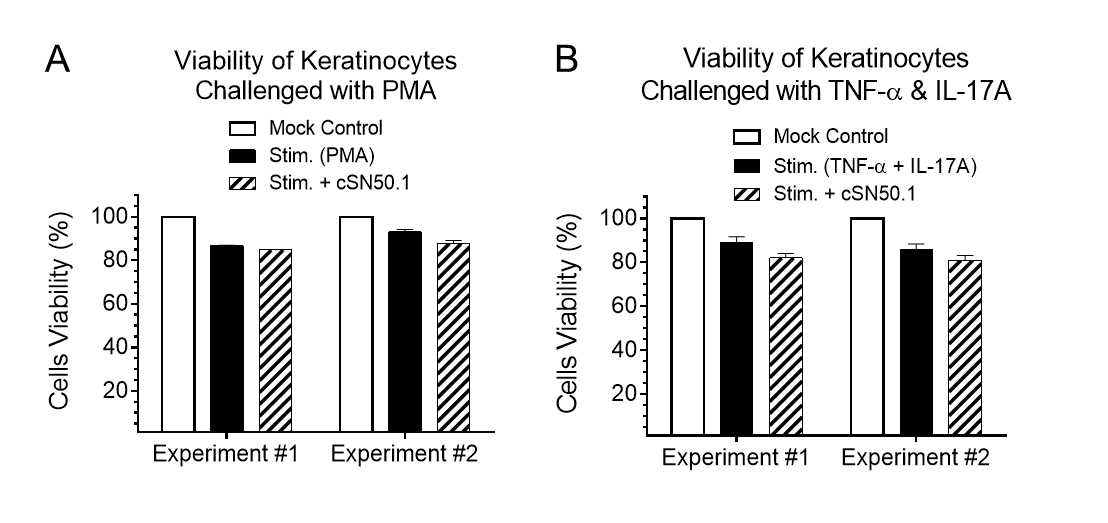
**

Keratinocytes were harvested at 24 hrs. (50 nM PMA challenge) or 48 hrs. (10 ng/ml TNF-α + 20 µg/ml IL-17A challenge). The cells viability was determined using trypan blue exclusion assay. A cells suspension was mixed 1:1 with 0.4% trypan blue and incubated for 3 min. at room temperature. The mixture was transferred to hemocytometer and counted manually. The viability is expressed as percentage of cells viable (clean cytosol) over total cells count. The data is presented as mean ± S.E.M. (n = 2).
